# Risk Assessment and Preventive Treatment for Peritoneal Recurrence Following Radical Resection for Gastric Cancer

**DOI:** 10.3389/fonc.2021.778152

**Published:** 2022-01-03

**Authors:** Lin Xiang, Shuai Jin, Peng Zheng, Ewetse Paul Maswikiti, Yang Yu, Lei Gao, Jing Zhang, Ying Zhang, Hao Chen

**Affiliations:** ^1^ The Second Clinical Medical College, Lanzhou University, Lanzhou, China; ^2^ Department of Pathology, Lanzhou University Second Hospital, Lanzhou, China; ^3^ Department of Technology, Beijing Weitai’an Pharmaceutical Ltd, Beijing, China; ^4^ Department of Laboratory Medicine, the First Medical Centre, Chinese PLA General Hospital, Beijing, China; ^5^ Department of Tumor Surgery, Lanzhou University Second Hospital, Lanzhou, China; ^6^ The Key Laboratory of the Digestive System Tumors of Gansu Province, Lanzhou University Second Hospital, Lanzhou, China

**Keywords:** gastric cancer, radical resection, peritoneal recurrence, risk assessment, preventive treatment

## Abstract

As the most common recurrence pattern after radical gastric cancer resection, peritoneal recurrence is a major cause of mortality, which affects the prognosis of patients to a very large extent. Peritoneal status and risk of peritoneal recurrence can be evaluated by peritoneal lavage cytology, photodynamic diagnosis, imaging examination, and pathologic analysis. Presently, there is no standard approach for preventing peritoneal recurrence after radical surgery; furthermore, controversies exist regarding the effects of some preventive methods. Among the preventive methods, there are high expectations about the potential of preoperative therapy, surgical skill improvement, hyperthermic intraperitoneal chemotherapy, and postoperative treatment to reduce the incidence of peritoneal recurrence after radical gastrectomy. This study aimed to analyze the results of previous studies on the risk assessment and preventive methods of peritoneal recurrence after radical gastrectomy in recent years. We hope to provide references for better approach to clinical diagnosis and treatment strategies for peritoneal recurrence after radical gastrectomy.

## Introduction

As a common malignant tumor of the digestive system, gastric cancer (GC) has the fifth highest incidence among malignant tumors worldwide and the third highest fatality rate, and there has been a significant increase in its incidence in East Asia (1). Currently, radical resection is the only curative treatment strategy for GC. However, many patients have recurrence after radical resection, and the prognosis of these patients is extremely poor. Furthermore, GC is mainly associated with the depth of tumor invasion, lymphatic involvement, and Borrmann type. The recurrence patterns after radical gastrectomy are classified as locoregional, peritoneal, and nonperitoneal distant recurrence. The most common site of first recurrence is the peritoneum (48.8%), then the liver (20.8%), and the locoregional (15.2%) (2). The significant reduction of survival time due to peritoneal recurrence is the leading cause of death (2–4). Therefore, early prevention and detection of recurrence with effective intervention are very important to improve the prognosis of patients with GC after radical resection. Presently, in patients with GC, many therapeutic methods and strategies have been used for the prevention and risk assessment of peritoneal recurrence after radical gastrectomy. To establish a reference value for the formulation of clinical strategies, this study aimed to identify current methods for predicting and preventing peritoneal recurrence after radical resection.

## Preoperatively

Before surgical operation, it is crucial to evaluate the risk of peritoneal recurrence and identify possible micrometastasis for appropriate treatment modality. Such evaluations can improve the accuracy of diagnoses, to ensure early intervention for high-risk patients, lead to the avoidance of unnecessary additional treatments for low-risk patients, and reduce additional harm from the redundant treatment of patients.

### Peritoneal Cytology

Intraperitoneal free cancer cells (IFCC) play a critical role in the development of peritoneal metastasis of GC (the main cause of failure after radical gastrectomy). Peritoneal lavage cytology, widely regarded as the gold standard for the diagnosis of IFCC, has negative and positive results reported as CY0 and CY1, respectively. Patients with positive peritoneal cytology have poor prognosis; therefore, a positive IFCC is considered an independent adverse prognostic factor. A retrospective review including GC patients with only CY1 status in the absence of obvious peritoneal metastasis reported that all patients had recurrence within 3 years after radical resection, and 92% of these patients had peritoneal metastasis, indicating positive cytology as an important precursor of peritoneal recurrence (5). Several previous studies have demonstrated that serosa infiltration is one of the most important predictors of peritoneal micrometastasis (6, 7). Furthermore, when serosa infiltration or suspected serosa infiltration occurs in GC patients, peritoneal lavage cytology should be implemented to confirm the existence of IFCC. However, GC patients with CY1 status are considered at stage IV, and their prognosis is still poor even after curative surgery for GC (8). Therefore, cytological examinations have a profound influence for GC patients in predicting peritoneal recurrence.

The detection methods of peritoneal cytology mainly include traditional cytology (hematoxylin and eosin staining, HE staining), immunoassay, immunohistochemistry (IHC), and reverse transcription polymerase chain reaction (RT-PCR). The accuracy, sensitivity, and specificity of predicting peritoneal recurrence differ and are 73–91.9, 11.1–80, and 86.4–100%; 72–95, 23–100, and 81–92.9; 54.8–76.7, 22.1–75, and 76.9–97.3%; and 61–89.7, 31–100, and 58.8–95% in traditional cytology, immunoassay, IHC, and RT-PCR, respectively (9). Compared with the remaining three methods, RT-PCR shows some advantages.

The main target of detection by RT-PCR is the carcinoembryonic antigen (CEA). In peritoneal lavage fluid, the sensitivity and diagnostic odds ratio of CEA protein or mRNA to predict peritoneal recurrence are higher than those of traditional cytology; however, traditional cytology has a higher specificity. GC CEA-positive patients are more likely to have peritoneal recurrence after radical resection, with significantly reduced overall survival (OS) (10). A meta-analysis of 117 cases with GC also had a similar conclusion (11). The peritoneal recurrence rate among patients with positive CEA was higher than that among negative-CEA patients. Furthermore, the expression of CEA in peritoneal lavage fluid was closely related to peritoneal recurrence after radical gastrectomy; this is considered the most important prognostic factor for recurrence after curative resection. Some scholars have suggested that the results of traditional cytology are so unstable that the detection of IFCC cannot be guaranteed, and it is necessary to combine them with those of other more sensitive molecular techniques (such as IHC or RT-PCR) to improve the detection rate of IFCC in the abdominal cavity (12).

Although RT-PCR shows advantages in accuracy, sensitivity, and specificity to IFCC detection, its procedure is cumbersome and time-consuming. It is impossible to provide reliable information to the surgeon during operation, which is a great limitation of practicability. The emergence of transcription-reverse transcription concerted reaction (TRC) seems to make up for the deficiency of RT-PCR. As a direct RNA amplification detection method, TRC was developed to detect peritoneal lavage fluid in GC patients (13). Moreover, compared with RT-PCR, TRC has a simpler operation maneuver and a faster detection strategy. The sensitivity (85%) and specificity (100%) of TRC are similar to those of RT-PCR (92 and 100%, respectively), but TRC is significantly faster and can be completed in 1.0–1.5 h (14). A prospective multicenter study of advanced GC (AGC) patients undergoing radical resection revealed that disease-free survival (DFS) and peritoneal recurrence-free survival (RFS) in the positive TRC-CEA group are significantly lower than those in the negative group and that TRC-CEA could be an important prognostic marker to predict survival and peritoneal recurrence in GC with serosal invasion (15). Another study showed that CEA detected by TRC after lymph node resection in radical gastrectomy is an important predictor of prognosis, although it is not closely related to peritoneal recurrence (16).

According to the above-mentioned studies, traditional cytology combined with other detection methods may be the best way to improve the detection rate of peritoneal cancer cells. This can help clinicians to identify the high-risk peritoneal recurrence groups and provide the key basis for the formulation of follow-up therapies.

### Imaging Examination

For GC, ^18^F-fluorodeoxyglucose positron emission tomography (^18^F-FDG PET) combined with computerized tomography (CT) (^18^F-FDG PET/CT) is often used to evaluate and predict recurrence prior to surgery and to monitor recurrence post-surgery, whereas its clinical significance has always been controversial in peritoneal recurrence prediction. A retrospective study involving 279 AGC patients who underwent ^18^F-FDG PET/CT before radical resection, with the tumor-to-normal liver uptake ratio (TLR) as the examination parameter, found a remarkably higher 5-year distant metastasis-free survival rate among patients with TLR ≤2.0 compared to that among patients with TLR >2.0 (95.5 *vs*. 68.8%, respectively, *P* < 0.0001); however, TLR had no significant correlation with peritoneal RFS (*P* = 0.7) (17). In addition, the attenuation of ^18^F-FDG uptake in the visceral adipose tissue (VAT) was found to be significantly associated with peritoneal RFS and OS, whereas in AGC patients with high VAT attenuation and a standardized uptake value (FDG uptake), peritoneal recurrence is more likely to occur after curative resection (18). In summary, depending on some specific parameters, preoperative PET/CT seems to be capable of being used to assess the risk of peritoneal recurrence after radical gastrectomy and may become an important non-invasive evaluation method. Since the sample size of this research was small and the finding was not very convincing, further studies with larger samples of clinical data to support these are still needed.

### Photodynamic Diagnosis

The diagnosis of peritoneal metastasis in patients with GC has a profound impact on treatment strategies. Currently, staging laparoscopy is routine in clinical settings; however, because some micrometastasis that are invisible to the naked eyes may be missed, eventually, this may lead to inappropriate radical resection in these GC patients. As a new technique for fluorescence imaging of lesions using photosensitive drugs, photodynamic diagnosis (PDD) shows great potential in the discovery of micrometastatic foci, with the commonly used drug being 5-aminolevulinic acid (ALA). ALA-PDD is more sensitive than white light laparoscopy in the detection of peritoneal dissemination, with an increased detection rate of peritoneal metastasis by 21–34% with ALA-PDD (19–21). If only white light observation is used without ALA-PDD detection, about 11% of patients with peritoneal dissemination will be missed, and most of these patients (76.9%) identified by ALA-PDD and confirmed to have peritoneal metastasis had negative cytological results (22). Although ALA-PDD shows obvious advantages in detecting peritoneal metastases that are not visible to the naked eye, its false positive rate is higher (32.3%) (23). ALA-PDD can improve the visualization of the invisible peritoneal metastases, and this helps to determine the peritoneal status of patients with AGC, resulting in GC staging accuracy. However, more large-sample randomized controlled clinical trials (RCTs) are needed to assess the applicability of PDD.

### Neoadjuvant Therapy

Neoadjuvant therapy could induce tumor downstaging and improve the rate of R0 resection for resectable GC (24). The neoadjuvant therapy methods are categorized as neoadjuvant chemotherapy (NAC), neoadjuvant radiotherapy, and neoadjuvant chemoradiotherapy. Radiotherapy is mostly used for esophagogastric junction cancer, while chemotherapy is mainly for GC. A meta-analysis involving 15 RCTs showed that neoadjuvant therapy could significantly reduce the overall mortality of AGC patients at 3 (relative risk, RR = 0.74, *P* = 0.005) and 5 (RR = 0.82, *P* = 0.009) years after radical surgery (25). Nonetheless, the postoperative recurrence pattern of patients who received NAC did not seem to have changed compared with the results of earlier studies, and the peritoneum was still the most common relapse site after radical gastrectomy in these patients (2, 26). Moreover, different types of NAC had no effect on 5-year RFS (*P* = 0.236). A retrospective study also reported no statistical difference in overall recurrence and peritoneal recurrence between NAC and surgery-only groups before or after a propensity score matching (27). Recently, the PRODIGY trial published the results indicating that adding NAC (docetaxel, oxaliplatin, and S-1) to the basic treatment of radical surgery plus S-1 adjuvant chemotherapy could notably improve progression-free survival in patients with resectable localized AGC (hazard ratio, HR = 0.70, *P* = 0.023), although there was no statistical difference in the OS (HR = 0.84, *P* = 0.338) (28). The detailed data related to recurrence were not reported. Surprisingly, some retrospective studies showed that neoadjuvant therapy before radical operation was an adverse factor for the long-term survival of AGC patients (HR = 1.631, *P* = 0.006) (2, 29). In terms of peritoneal recurrence, the proportion of patients receiving neoadjuvant therapy was even higher than that of untreated ones (37.1 *vs*. 32.1%). The above-mentioned conditions may occur because patients who receive neoadjuvant therapy tend to have relatively more advanced stage or high-grade tumors, which may explain the difference between pTNM stage and ypTNM stage (post-neoadjuvant pTNM).

Although many studies have concluded that neoadjuvant therapy has no effect on peritoneal recurrence in GC patients after radical resection (2, 26, 27, 29, 30), the recent findings by Xu et al. seem to have reinforced the confidence in neoadjuvant therapy to reduce peritoneal recurrence (31). By propensity score-matched analysis, Xu et al. found that, for local AGC with serosal invasion, the OS and DFS in NAC-treated patients were significantly better than that in the untreated ones (*P* < 0.0001), and patients who received NAC had fewer postoperative complications (*P* = 0.037). It is exciting to note that the overall recurrence in the NAC group was less than that in the non-NAC group (29.9 *vs*. 63.3%), and peritoneal recurrence significantly decreased (19.0 *vs*. 48.4%). According to the above-mentioned studies, NAC appears to have the potential to improve prognosis and prevent recurrence, especially for patients who are at a high risk of peritoneal recurrence, including serosa-positive patients. Neoadjuvant therapy is recommended for T ≥3 and/or with node-positive GC, according to The Italian Research Group for Gastric Cancer (32). A multicenter randomized phase II trial (NCT02931890) is underway to explore different neoadjuvant therapy regimens (chemotherapy, chemotherapy followed by chemoradiotherapy, and chemoradiotherapy) for the purpose of identifying a comprehensive and objective clinical evaluation (33). At present, the populations among whom neoadjuvant therapy is being implemented differ between Eastern and Western countries, and no consensus has been reached yet. However, most studies set the treatment range to patients with T ≥3 tumor and N+ tumor.

After diagnosis and staging by routine endoscopy examination, endoscopic biopsy, and contrast-enhanced CT, laparoscopy with/without peritoneal lavage cytology is recommended for patients with stage I B or higher GC or with suspected peritoneal metastasis (32, 34, 35). For GC patients with positive peritoneal cytology or macroscopic peritoneal metastases, it is necessary to change the treatment strategy instead of direct radical operation. Preoperative chemotherapy is needed to improve the possibility of R0 resection to avoid incomplete resection and reduce the risk of cell peritoneal seeding during surgery.

## Intraoperatively

### Surgical Maneuver

In addition to the serosa infiltration of gastric tumors which increases the risk of peritoneal dissemination, surgical procedures may also cause cancer cells to enter and penetrate the abdominal cavity from the resection margin, blood, or lymphatic vessels and eventually lead to peritoneal metastasis. Radical gastrectomy includes open surgery, laparoscopic surgery, robotic surgery, and endoscopic procedure; among these, endoscopic procedure is mainly aimed at local early-stage GC. According to the cytological analysis of peritoneal lavage in GC patients undergoing radical resection, the diffusion of tumor cells into the peritoneal cavity after operation is higher than that when the abdominal cavity has just been opened and explored, which suggest that the operation could directly promote the iatrogenic dissemination of tumor cells and increase the possibility of peritoneal metastasis (36). Therefore, surgical methods and related precautions for GC have become important concerns for clinicians to reduce postoperative peritoneal recurrence.

Due to extensive trauma, poor postoperative recovery, and other complications, the traditional open radical resection of GC is rapidly giving way to minimally invasive surgery (MIS). Furthermore, a large number of studies have shown that laparoscopic radical gastrectomy has comparable short- and long-term outcomes compared to traditional open radical gastrectomy and is suitable at all stages for GC curative purposes (37–42). According to these literatures, there is no consensus on whether MIS is superior to open surgery in the short- and long-term outcomes, but MIS is, at least, not inferior to traditional open surgery. A propensity score−matched analysis from an eastern center concluded that the postoperative complications (35.2 *vs*. 40.7%, *P* = 0.69) and 90-day mortality (1.9 *vs*. 3.7%, *P*=1.00) in the laparoscopic gastrectomy (LG) group were comparable to those in the open gastrectomy (OG) group (42). Although there was no significant difference between the two groups in 3-year OS and DFS (*P* = 0.34; *P* = 0.51), the LG group had markedly fewer peritoneal recurrences than the OG group (3.7 *vs*. 27.8, *P* < 0.01). Another recent propensity score−matched analysis from a western center also reported similar results of no statistical difference in OS, DFS, postoperative complications, and mortality between LG and OG groups (43).

Compared with open resection, the postoperative overall recurrence of laparoscopic gastrectomy has limited demerits, and its peritoneal recurrence rate is not higher than that of traditional open surgery (39, 41, 44). KLASS-01, a large RCT, showed that, for patients with clinical stage I GC, the long-term oncological outcomes of laparoscopic distal gastrectomy (LDG) and open distal gastrectomy (ODG) are similar, with no significant difference in peritoneal recurrence between them (1.2 *vs*. 1.0%) (45). Furthermore, the other two RCTs (CLASS-01 and KLASS-02) for locally AGC also achieved similar results with similar 3-year DFS in the LDG and ODG groups and no significant difference between the two groups in peritoneal recurrence (46, 47). Shi et al. studied the long-term tumor outcomes of patients with locally AGC after radical resection and found that the 5-year OS and DFS do not notably differ between the LG and OG groups, with no statistical difference in peritoneal recurrence (LG: 28.6% *vs*. OG: 26.0%, *P* = 0.705) and other types of recurrence, between the two groups (48). Based on the above-mentioned research, laparoscopic radical gastrectomy could achieve short- and long-term outcomes comparable to open surgery while not increasing the probability of peritoneal recurrence but showing significant and more prominent advantages in other aspects, such as reduced intraoperative blood loss and early postoperative recovery. Compared with laparoscopic surgery, robotic surgery, another MIS, has prominent advantages, such as fatigue reduction, high stability, and three-dimensional vision, and has been gradually applied in the treatment of GC. In addition, many studies have also reported better minimally invasive advantages with robotic surgery than laparoscopic surgery in radical gastrectomy, and these two operative methods have similar short- and long-term outcomes as well as postoperative peritoneal recurrence rates (49–53). Regarding the high expense of robotic surgery, its application in the treatment of GC is still not yet popularized. Therefore, laparoscopic resection has gradually replaced traditional open resection to become the mainstream method of radical gastrectomy.

Lymph node dissection is an important part of radical gastrectomy and usually classified as D1, D2, and D3 lymphadenectomy according to the extent of dissection. For resectable GC, D2 lymphatic dissection is mainly recommended (54). Currently, there is insufficient and effective evidence for the relationship between the extent of lymph node dissection and peritoneal recurrence after radical gastrectomy. In the Dutch D1D2 trial, despite the absence of noticeable differences in 15-year OS, DFS, and relapse rate between D1 and D2 groups, the cancer-related mortality rate in D1 group was higher than that in D2 group (48 *vs*. 37%, *P* = 0.01) (55). The patients who received D1 lymphadenectomy showed higher rates of locoregional and liver recurrences than those undergoing D2 lymphadenectomy, but the data related to peritoneal recurrence were not reported in this publication. In another research, Nakanishi et al. found no significant difference in 5-year cumulative peritoneal recurrence rates between D2 minus and D2 groups for AGC patients with CY0 (29 *vs*. 33%, *P* = 0.595) (56). A retrospective study involving 568 AGC patients reported that the overall recurrences of D2 and D3 patients are comparable (57). Furthermore, there was no statistical difference in peritoneal recurrence rates (14.6 *vs*. 11.6%, *P* = 0.319) and other types of recurrence between the two groups. Similarly, a recently published retrospective cohort analysis reported comparable rates of peritoneal recurrence in the D1 plus and D2 groups (4.4 *vs*. 5.0%, *P* = 0.743) (58). According to the above-mentioned studies, the extent of lymph node dissection during radical resection of GC seems to have no correlation with postoperative peritoneal recurrence. Nevertheless, the propensity score-matched analysis by Hayashi et al. provides some interesting results (59). They reported that the number of retrieved lymph nodes (RLN) is related to the long-term outcome of AGC patients after radical surgery. The RLN ≥40 group had notably longer OS and RFS than the RLN <40 group (HR = 2.11, *P* = 0.0057; HR = 2.35, *P* = 0.0001). Furthermore, compared with the RLN ≥40 group, the peritoneal recurrence rate in the RLN <40 group increased significantly (*P* = 0.0007).

As treatment strategies to prevent peritoneal metastasis after radical gastrectomy, the use of either omentectomy or bursectomy has always been controversial. A multicenter prospective cohort study showed that the incidence of omentum metastasis in curable GC is lower and is only related to later clinical stage and non-curable features, and it suggested that omentum resection is not necessary in radical gastrectomy (60). Sakimura et al. reported that, for patients with AGC who underwent radical resection, there was no significant difference between the omentectomy and non-omentectomy groups in 3-year OS and RFS as well as in overall and peritoneal recurrence rates, which suggest that omentectomy could not improve the survival benefits of AGC patients (61). According to some data from an earlier RCT, the peritoneal recurrence in patients who underwent radical gastrectomy plus bursectomy was less than that of those without bursectomy (8.7 *vs*. 13.2%). Although the 3-year OS in the bursectomy group was better than that in the non-bursectomy group, there was no statistically significant difference between groups (62). A subsequent large retrospective study found that, for AGC patients that underwent radical surgery, additional bursectomy had no significant effect on the OS rate (*P* = 0.978), and there was no significant difference in peritoneal recurrence between the bursectomy and non-bursectomy groups (*P* = 0.623) (63). In 2018, a phase 3, open-label RCT (JCOG1001) that explored the survival benefit of bursectomy for resectable GC was published. The 5-year OS in the non-bursectomy group (omentectomy alone) was 76.7%, compared with 76.9% in the bursectomy group (one-sided *P* = 0.65), with no extra survival benefit from bursectomy. Moreover, based on the JCOG1001 data, the peritoneal recurrence rate in the bursectomy group was also the same as that in the non-bursectomy group (44%), suggesting that bursectomy could not improve peritoneal recurrence (64). In the light of the above-mentioned studies, omentectomy and bursectomy not only failed to prevent peritoneal metastasis or to improve the long-term survival but also increased the operation time, intraoperative blood loss, and complications. Therefore, it seems meaningless to add omentectomy or bursectomy to the radical resection of GC. Although many studies have reported that omentectomy does not improve survival benefits to patients, it is still part of the standard gastrectomy guidelines (32, 35, 65). This may be because it is easier to perform omentectomy than preserve the omentum in GC resection, and omentectomy is beneficial for lymph node dissection. Bursectomy is mainly used in Japan and included in Japanese GC treatment guidelines (65). The fifth edition of the Japanese guidelines refers to the conclusion of the JCOG1001 trial, but bursectomy has not been revised yet (64, 65). The sixth edition of the Japanese guidelines may reinterpret the application of omentectomy and bursectomy.

In the process of radical surgery for GC, blood from intraoperative bleeding easily accumulates in the abdominal cavity, which brings the peritoneal surface directly in contact with blood components, thus activating the extravascular blood cells to produce a variety of cytokines and thereby providing a favorable survival microenvironment for tumor cells that leak into the abdominal cavity. A retrospective study of 540 patients with AGC who underwent radical resection found that large intraoperative bleeding is associated with a high risk of peritoneal metastasis, whereas small bleeding is not, and patients with large intraoperative hemorrhages are more likely to develop peritoneal recurrence (66). Another retrospective research showed a significantly higher peritoneal recurrence rate in patients who received allogeneic blood transfusion during the perioperative period of radical resection for GC than that in patients without allogeneic blood transfusion (22.8 *vs*. 9.3%); however, the rates of metastasis to the liver, lung, and lymph nodes did not change (67). Therefore, surgeons should avoid the higher risks of postoperative peritoneal recurrence related to the development of intraoperative bleeding by minimizing intraoperative blood loss to avoid allogeneic blood transfusion.

### Extensive Intra-Operative Peritoneal Lavage

The mechanism of extensive intra-operative peritoneal lavage (EIPL), a simple adjunctive surgical method, is based on limited dilution to reduce the risk of cancer cell dissemination resulting from surgery. Previous studies have shown that EIPL could effectively reduce the level of cancer cells spreading in the peritoneal cavity during radical resection for GC, and the use of distilled water is as effective as normal saline (36). However, the CCOG1102 trial showed the opposite results of no significant difference in peritoneal relapse-free survival rate (*P* = 0.676) and DFS and OS between the EIPL and non-EIPL groups after radical gastrectomy. In this trial, EIPL could neither reduce postoperative peritoneal dissemination nor improve the prognosis of patients, but it seemed to ameliorate DFS for patients with higher intraoperative blood loss or postoperative abdominal infection (68). In this study, most patients had negative peritoneal cytology results, so, even if there were undetected free tumor cells, washing the abdominal cavity with less saline is sufficient to remove them in the non-EIPL group, which may be the reason why there was no significant difference between the EIPL and non-EIPL groups. In the latest phase 3, multicenter, large-sample RCT (NCT02140034), the 3-year OS rates in the EIPL and surgery-alone groups were 77.0 and 76.7% (*P* = 0.62), respectively, while the 3-year cumulative incidences in peritoneal recurrence were 7.9 and 6.6% (*P* = 0.35), respectively. On the contrary, EIPL not only failed to reduce peritoneal recurrence and improve patient survival but also significantly increased the incidence of adverse events (69). Wound infections and liver function abnormalities were more common in patients receiving EIPL than in patients undergoing surgery alone (2.0 *vs*. 0.3% and 1.7 *vs*. 0.3%, respectively). Furthermore, the incidence of death due to adverse events in the EIPL group (2.3%) was also higher than that in the surgery-alone group (0.6%). However, another large-scale, multicenter (11 centers) RCT (NCT02745509) in China showed that the postoperative adverse events and mortality in the EIPL group are lower than those of the surgery-alone group (11.1 *vs*. 17.0%, *P* = 0.04; 0 *vs*. 1.9%, *P* = 0.02) (70). In this trial, the long-term results have not been released yet.

### Hyperthermic Intraperitoneal Chemotherapy

Hyperthermic intraperitoneal chemotherapy (HIPEC), a combination therapy with precise temperature control for circulating intraperitoneal perfusion of chemotherapeutic agents, has been widely used in the prevention and treatment of peritoneal metastatic tumors. For patients with serosal invasion in GC, compared with the non-HIPEC group, the survival of HIPEC patients significantly improved (*P* < 0.00001), with a remarkably reduced peritoneal recurrence rate (*P* = 0.001) (71). A retrospective study involving 38 GC patients with serosal invasion showed that the peritoneal recurrence in the HIPEC group is dramatically lower than that in the radical-surgery-alone group (11.1 *vs*. 73.7%, respectively, *P* < 0.001) (72). In another RCT of resectable AGC, the results were similar to the previous RCT results, with much higher peritoneal recurrence in the non-HIPEC group (30%) than that in the HIPEC group (5%) (73). Taken together, HIPEC is an effective method for preventing postoperative peritoneal metastasis in high-risk patients. Presently, the use of HIPEC in most countries is mainly confined to the treatment of peritoneal metastatic carcinoma, which has not been included as a standard practice for the preventive therapy of peritoneal recurrence. Firstly, there may be few medical institutions with equipment and conditions for such treatment. Secondly, the pros and cons of whether the conditions of the patients would still be conducive to accepting HIPEC after severe trauma from radical surgery need to be weighed. Most importantly, there is still a lack of valid evidence from large-sample-size RCTs to support the survival benefits of HIPEC.

## Postoperatively

### Pathological Analysis

In addition to the previously mentioned methods for evaluating the risk of preoperative peritoneal recurrence, pathological analysis of the resected tumor specimens also has considerable value. In AGC patients undergoing radical gastrectomy, the depth of tumor invasion was the only risk factor significantly associated with peritoneal recurrence (74). Yoo et al. analyzed the prospective data of 655 patients that underwent radical resection for GC. The time to peritoneal relapse in patients with macroscopic serosal lesions was considerably shorter than that in patients without serosal lesions (*P* < 0.001), and the 5-year peritoneal recurrence rates were 32.8 and 8.7%, respectively. These results suggest that the macroscopic assessment of serosal lesions may be a useful index to predict the risk of peritoneal recurrence after radical resection (75). In accordance with the Japanese Classification of Gastric Carcinoma, tumor infiltrative pattern (INF) is classified into INFa (expanding growth with a distinct border from the surrounding tissue), INFb (an intermediate pattern between INFa and INFc), and INFc (infiltrative growth with no distinct border with the surrounding tissue) (76). Compared with INFa and INFb, INFc patients had significantly more peritoneal metastases; furthermore, INF was found to be an independent risk factor for peritoneal recurrence after radical gastrectomy (77–80). A previous study found that adjuvant chemotherapy could not improve the peritoneal recurrence rate in INFc group but reduced the rate in the INFa/b group (78). In a recent study, Chen et al. employed multiphoton imaging technology to quantitatively analyze the collagen characteristics in the tumor microenvironment from the tissue specimens infiltrating the gastric serosa. They revealed that the features of collagen are related to postoperative peritoneal metastasis in GC with serosal invasion. Furthermore, a collagen nomogram that they constructed to predict the risk of peritoneal recurrence with serosa-positive GC after radical gastrectomy displayed a stronger predictive power than the clinicopathological model (81).

### Early Postoperative Intraperitoneal Chemotherapy

To eliminate the microscopic peritoneal lesions after resection of GC, early postoperative intraperitoneal chemotherapy (EPIC) is administered using mitomycin C and 5-fluorouracil (5-FU) or taxanes through inflow and outflow catheters. This is usually performed 1–5 days after surgery and then repeated subsequently every 24 h. As reported in another study, although the safety of EPIC after radical gastrectomy was acceptable, there was no difference in postoperative survival between patients who received EPIC and those who did not, implying that EPIC could not provide survival benefits to patients undergoing curative resection for GC (82). However, the small sample size of 46 patients was a limitation of that study. A retrospective study based on 245 serosa-positive GC patients who underwent radical surgery found that the 5-year OS- and GC-specific survival rates in the EPIC group were significantly better than those in the non-EPIC group. Moreover, the rate of peritoneal recurrence in the EPIC group is notably lower than in the non-EPIC group (18.5 *vs*. 32.2%, *P* = 0.038) (83). Therefore, EPIC appears to be an effective method for GC patients at a high risk of peritoneal recurrence by improving their survival through reducing peritoneal metastases.

### Adjuvant Chemotherapy

Systemic adjuvant chemotherapy for GC patients after surgery has always been important in clinical studies, and clinicians pay great attention to the formulation of a therapeutic regimen and the effects of different schemes on postoperative recurrence. Adjuvant chemotherapy regimens generally include oral 5-FU as monotherapy (including S-1 and capecitabine) or combined with oxaliplatin. In a phase 2 clinical trial (CCOG0301), the 2-year survival rate was higher among GC patients with positive peritoneal lavage cytology treated with oral S-1 after radical resection than among historical controls (84). All the cases included in this trial had positive peritoneal cytology. Sasako et al. conducted a randomized phase 3 clinical trial to evaluate the effect of S-1 as adjuvant chemotherapy for GC patients after radical resection. They found that the 5-year survival outcomes in the S-1 group are better than that in the surgery-only group. In terms of recurrence, the overall relapse rate in the S-1 group is lower than that in the surgery-only group; in particular, the lymph nodes and peritoneum recurrence rates decreased significantly, and postoperative adjuvant therapy with S-1 could reduce the risk of recurrence by 34.7% (85). The patients enrolled in the study had pathological stage II or IIIA/B with CY0, and the chemotherapy regimen was still S-1 monotherapy. In another phase 3 clinical trial, JCOG9206-2, cisplatin combined with UFT could not improve the overall and relapse-free survival in patients with serosal invasive GC after radical resection (86). According to the data in this trial, adding adjuvant chemotherapy, compared with surgery alone, could not reduce peritoneal metastasis. The patients who participated in the trial had GC with macroscopic serosa-invasive, negative peritoneal lavage cytology without distant metastasis. The postoperative treatment plan was intraperitoneal chemotherapy with cisplatin before abdominal closure, followed by intravenous chemotherapy (cisplatin + 5-FU + UFT). A meta-analysis including 3,897 patients undergoing radical resection of GC showed that the addition of adjuvant chemotherapy significantly reduced the rate of peritoneal recurrence compared with surgery alone (*P* = 0.001) (87). However, the above-mentioned research only analyzed all the regimens together and did not classify the specific adjuvant chemotherapy schemes in the analyses. Recently, a randomized, controlled phase 3 trial of S-l plus docetaxel adjuvant chemotherapy in patients after radical gastrectomy reported that the 3-year RFS in the S-l plus docetaxel group was better than that in the S-1 monotherapy group (*P* < 0.001). Although the hematogenous site and node recurrence rates in the combination group are significantly lower than that in the S-1-alone group, there was no statistically significant difference between these two groups in peritoneal relapse (9.3 *vs*. 12.9%, *P* = 0.092). This suggests that S-1, combined with docetaxel adjuvant chemotherapy regimen, does not improve peritoneal metastasis after radical resection of GC compared with S-1 monotherapy (88).

In accordance with previous studies, there is yet no definite conclusion on the effects of postoperative adjuvant chemotherapy in the prevention of peritoneal metastasis after radical gastrectomy of GC. Drug selection and therapeutic regimen are crucial for the appropriate method, and this still needs to be supported with a large number of clinical studies.

## Conclusions

The occurrence of peritoneal metastasis after radical gastrectomy seriously affects the prognosis of patients to a great extent, and how to identify patients at a high risk of peritoneal recurrence and develop preventive treatment approaches quickly is vital for the reduction of postoperative peritoneal metastasis. The progression degree of GC is significantly correlated with the resection effect of radical gastrectomy. The greater the progression degree, the lower the possibility of R0 resection that is accompanied by patients at a high risk of peritoneal metastasis. Even if patients with AGC could receive curative surgery and other related treatments, their long-term survival is still relatively poor. Undoubtedly, early detection of tumors cannot only improve the effect of radical resection but also greatly reduce the risk of spread of tumor cells in the peritoneal cavity during operation. Therefore, cancer screening has a more practical significance than any other therapeutic method to avoid postoperative peritoneal metastasis. Presently, many countries with a high incidence of GC, such as Japan and South Korea, have established and formulated their own guidelines for GC screening. There are certain effects on preventing peritoneal metastasis after radical resection of GC through accurate judgment of determining peritoneal status, improvement of surgical procedure, peritoneal lavage, intraperitoneal chemotherapy, and adjuvant chemotherapy (**Tables 1**, **2**). Despite all these findings, several prospective multicenter studies are essential to elucidate clinical evidence, promoting the criteria for the prevention of peritoneal recurrence after radical resection in the management of GC.

**Table 1:** Predictive methods of peritoneal recurrence.

Timing	Method		Potential clinical value
Preoperatively	Peritoneal lavage cytology	Traditional cytology (HE staining)	The most important risk predictor Traditional cytology combined with molecular biology techniques such as RT-PCR could improve the detection effect TRC could significantly improve the detection efficiency
		Immunoassay	
		Immunohistochemistry	
		RT-PCR	
		TRC	
	^18^F-FDG PET/CT		Simple and feasible; limited reference value and lack of evidence
	Photodynamic diagnosis		Improve the detection rate of micrometastases and make the staging more accurate, but the false positive rate is high
Postoperatively	Pathological analysis		An important risk predictor of peritoneal recurrence; high feasibility and reference value

**Table 2:** Preventive methods of peritoneal recurrence.

Timing	Method		Potential clinical value
Preoperatively	Neoadjuvant therapy		Certain preventive value for patients at stage T≥3 and N+
Intraoperatively	Surgical maneuver	Open gastrectomy	No significant difference in peritoneal recurrence among the three surgical methods Considering the intraoperative blood loss and postoperative recovery, laparoscopic resection could be actively selected
		Laparoscopic gastrectomy	
		Robotic gastrectomy	
		Lymphadenectomy	No definite relevance between the extent of dissection and peritoneal recurrence
		Omentectomy and bursectomy	Be cautious to use bursectomy
		Others	Reducing the amount of intraoperative blood loss could diminish the risk of peritoneal recurrence
	EIPL		It could be considered for patients with massive intraoperative blood loss, while conventional peritoneal lavage for the rest
	HIPEC		Could be considered for patients with a high risk of peritoneal recurrence and permitting physical conditions
Postoperatively	EPIC		Could be considered for patients with a high risk of peritoneal recurrence
	Adjuvant chemotherapy		Doubt in preventive effect

## Author Contributions

LX and HC contributed to the conception and design of the review. LX and SJ collected the related paper and drafted the manuscript. YZ, PZ and EM revised the manuscript. All authors contributed to the article and approved the submitted version.

## Funding

This work was supported by fund from the National Key Research and Development Program of China (2017YFC1601502); Key Talents Project of Gansu Province (2019RCXM020); Key Project of Science and Technology in Gansu Province (19ZD2WA001); Science and Technology Project of Chengguan District of Lanzhou City (2019RCCX0034); Cuiying Scientific and Technological Innovation Program of Lanzhou University Second Hospital (CY2017-ZD01); and Project supported by the Science-Technology Foundation for Young Scientist of Gansu Province (21JR1RA163).

## Conflict of Interest

Author SJ was employed by company Beijing Weitai’an Pharmaceutical Ltd.

The remaining authors declare that the research was conducted in the absence of any commercial or financial relationships that could be construed as a potential conflict of interest.

## Publisher’s Note

All claims expressed in this article are solely those of the authors and do not necessarily represent those of their affiliated organizations, or those of the publisher, the editors and the reviewers. Any product that may be evaluated in this article, or claim that may be made by its manufacturer, is not guaranteed or endorsed by the publisher.
